# HLA‐B*44 Alleles and HLA‐DQA1*03:01 as Genetic Risk Factors for Drug‐Induced Liver Injury due to Fluoroquinolones

**DOI:** 10.1111/liv.70699

**Published:** 2026-05-15

**Authors:** Paola Nicoletti, M. Isabel Lucena, Raul J. Andrade, Samreen Zafer, Einar S. Bjornsson, Pär Hallberg, Dominique Larrey, Mariam Molokhia, Mia Wadelius, Guruprasad P. Aithal, Ann K. Daly

**Affiliations:** ^1^ Department of Genetics and Genomic Sciences Icahn School of Medicine at Mount Sinai New York USA; ^2^ UGC Digestivo y Servicio de Farmacología Clínica, Instituto de Investigación Biomédica de Málaga‐Plataforma Bionand (IBIMA Plataforma Bionand), Hospital Universitario Virgen de la Victoria Universidad de Málaga Málaga Spain; ^3^ Centro de Investigación Biomédica en Red de Enfermedades Hepáticas y Digestivas (CIBERehd) Madrid Spain; ^4^ Division of Gastroenterology and Hepatology, Department of Internal Medicine The National University Hospital of Iceland Reykjavik Iceland; ^5^ Faculty of Medicine University of Iceland Reykjavik Iceland; ^6^ Department of Medical Sciences, Clinical Pharmacogenomics Uppsala University Uppsala Sweden; ^7^ Department of Hepato‐Gastroenterology CHU Montpellier, Montpellier University/INSERM U1183 Montpellier France; ^8^ School of Life Course & Population Sciences, Faculty of Life Sciences and Medicine King's College London UK; ^9^ NIHR Nottingham Biomedical Research Centre Nottingham University Hospitals NHS Trust and University of Nottingham Nottingham UK; ^10^ Translational & Clinical Research Institute, Newcastle University, Faculty of Medical Sciences Newcastle upon Tyne UK

**Keywords:** ciprofloxacin, drug‐induced liver injury, fluoroquinolone, HLA allele

## Abstract

**Background and Aims:**

Fluoroquinolone exposure can give rise to drug‐induced liver injury. Genetic risk factors for this form of liver injury are poorly understood but some evidence suggests that HLA genes contribute. This study aimed to characterise HLA risk factors for fluoroquinolone‐induced liver injury in a European population.

**Methods:**

From the previously described iDILIC cohort, 22 cases of fluoroquinolone‐induced liver injury were identified, with most cases (*n* = 13) related to ciprofloxacin. Liver injury cases due to other drugs (*n* = 458), also from iDILIC, served as controls. HLA genotypes were imputed from genome‐wide association analysis data using SNPtoHLA. Frequency differences between cases and controls for HLA alleles, HLA amino acids and HLA families were assessed by applying a logistic regression model.

**Results:**

HLA‐significant associations were detected for HLA class I and II alleles with individual allelic associations detected for HLA‐B*44:03, HLA‐A*29:02, HLA‐C*16:01, HLA‐DQA1*03:01 and several *HLA‐DRB1* alleles. Combined HLA‐B*44 alleles were associated with DILI with an odds ratio of 5.33 [95% confidence interval 2.45–11.61; *p* = 2.5 × 10^−5^]. Serine at position‐167 in HLA‐B, specific to B*44:02, B*44:03 and B*45:01, was associated with a significantly increased risk of liver injury (*p* = 4.7 × 10^−6^), but no other HLA‐B amino acids were significant. The B*44 association was also significant among ciprofloxacin cases only.

**Conclusions:**

A novel HLA‐B*44 association with fluoroquinolone‐induced liver injury has been identified and a previously reported but weaker association with HLA‐DQA1*03:01 confirmed. The B*44 findings suggest a mechanism involving inappropriate recognition of drug‐modified self peptides by serine‐167 in pocket A of the HLA‐B protein.

## Lay Summary

Fluoroquinolone antimicrobials may cause drug‐induced liver injury. This study describes the clinical characteristics of a European cohort involving 22 cases who developed drug‐induced liver injury after exposure to various fluoroquinolones. Certain HLA genotypes increase the risk of developing this adverse drug reaction.

**Abbreviations**
AF: allele frequency
DILI: drug‐induced liver injury
FQ: Fluoroquinolone FQ
GWAS: genome‐wide association study
HLA: human leukocyte antigen
iDILIC: International Drug‐Induced Liver Injury Consortium
MHC: major histocompatibility complex
OR: odds ratio
PC: principal component
RUCAM: Roussel Uclaf Causality Assessment Method
SNP: single nucleotide polymorphism
USA NMDP: USA National Marrow Donor Program

## 1 Introduction

Idiosyncratic drug‐induced liver injury (DILI) is rare but can have serious consequences. Genetic risk factors for DILI have been studied extensively, with the strongest associations seen for particular HLA genes [1]. Despite these common HLA associations, DILI reactions are not necessarily considered an immune reaction and non‐HLA genes may also contribute [2]. The majority of the HLA associations are with HLA class I genes, though a few well‐established associations with HLA class II also occur [1]. In parallel with the reports on HLA associations, attempts have been made to understand the underlying mechanism for these associations. The overall data is generally consistent with an inappropriate T cell response to drug‐protein or metabolite‐protein complexes [3]. In the growing list of HLA associations with DILI due to specific drugs, some very specific allele‐drug associations have been reported. Occasionally, associations between particular alleles and several drugs with chemically diverse structures have been observed [1].

The fluoroquinolones (FQ) are a group of broad‐spectrum antimicrobials, used widely since the 1980s. They were developed by modification of the older quinolones, including addition of a fluorine [4]. Despite their development being generally considered a valuable advance initially, it is now clear that there are also problems with adverse reactions to these drugs which have led to recommendations restricting their use [5]. The adverse reactions particularly involve damage to tendons, muscle and the nervous system but FQ‐induced liver injury cases have also been reported [6, 7, 8, 9, 10, 11]. The risk of DILI with FQs appears to extend to several currently licensed members of this drug class and is estimated to be 5.0–9.9 events per 10 000 person‐years, comparable to that seen for amoxicillin‐clavulanate [10].

A number of cases of DILI related to FQs have been included in previous studies on DILI genetic risk factors [12, 13, 14] but no specific genome‐wide significant associations were detected, probably due to limited numbers with this form of DILI. A recent study of a separate FQ‐DILI cohort from the USA reports some novel associations with HLA‐DQA1*03:01 and HLA‐B*57:01 [15]. To extend these findings, we have undertaken a more detailed analysis of HLA alleles in FQ‐DILI cases recruited from European centres as part of the iDILIC study [13]. In addition to considering individual HLA class I and II alleles as genetic risk factors, we also consider HLA‐B alleles as a larger subgroup, allowing key amino acid residues to be assessed as DILI risk factors.

## 2 Materials and Methods

### 2.1 Enrolment Details

Cases and the main control group were derived from the iDILIC study. As described in detail previously, all participants included in iDILIC provided written informed consent, and each study was approved by the appropriate national or institutional ethical review boards [13].

### 2.2 Causality Assessment

DILI cases were evaluated by application of the Council for International Organisations of Medical Science (CIOMS) scale, also called the Roussel Uclaf Causality Assessment Method (RUCAM) [16] and by expert hepatologist review. The pattern and severity of liver injury were classified according to the International Consensus Meeting Criteria [17]. Only cases having at least a possible causality (score ≥ 3) were included in the study.

### 2.3 Cases and Controls

We studied clinical features and HLA genotypes of 22 European DILI cases due to any of the fluoroquinolones. These cases have been reported previously elsewhere [13].

To allow for population matching, the main control group used consisted of European DILI cases (*n* = 458) where the causative drug for DILI was not a FQ, flucloxacillin or amoxicillin‐clavulanate. Cases relating to flucloxacillin and amoxicillin‐clavulanate were not included due to well‐established HLA risk factors for DILI due to these drugs. A list of all causative drugs in the control group is provided in Table S1. As shown in Figure S1, principal component analysis (PCA) demonstrated genetic matching between cases and controls.

To evaluate whether allele frequencies in DILI controls were similar to those of the general population, we compared them with allele frequencies reported for the USA NMDP European Caucasian cohort (*n* = 1 242 890) on the HLA allele frequencies database at www.allelefrequency.net.

### 2.4 DNA Preparation and Genome‐Wide Genotyping

DNA preparation and genome‐wide genotyping on the Illumina IM and Infinium HumanCoreExome BeadChip were as described previously [13].

### 2.5 SNP and HLA Imputation

SNP imputation was performed as described previously [13]. Four‐ and two‐digit HLA alleles and amino acid changes (AA) were also inferred using SNP2HLA using reference data collected by the Type 1 Diabetes Genetics Consortium (T1DGC) [18].

### 2.6 Statistical Analysis

All genetic analyses were performed as described previously [13]. Briefly, genetic ancestry and corresponding principal components were imputed by EIGENSTRAT. Then, we tested for the differences in allele frequency of (a) each HLA allele, (b) amino acid (AA), and (c) HLA family between cases and controls, applying a logistic regression model, adjusting for age and sex and two significant principal component axes. MHC significance was defined using the Bonferroni correction threshold of *p* < 0.0002 (0.05/200, accounting for 200 observed HLA alleles) for alleles *p* < 0.004 (for 103 observed HLA families) and *p* < 0.00005 (for 1133 observed AA). Conditional analyses in the MHC region were undertaken, and the genotypes at the conditioning marker(s) were included as covariates under an additive model. To assess the general similarity of peptide binding of HLA B alleles without knowledge of immunogenic peptide(s), we used MHCcluster 2.0 [19].

## 3 Results

### 3.1 Characteristics of the Study Cases and Controls

The FQ‐DILI cases in the study were from two separate recruitment phases of the iDILIC study [13]. Phase I consisted of two cases from the Eudragene network included in an earlier study [12] and phase II 20 cases recruited during the iDILIC study [13]. The combined 22 DILI cases were due to ciprofloxacin (*n* = 13), levofloxacin (*n* = 4), moxifloxacin (*n* = 3), norfloxacin (*n* = 1) and trovafloxacin (*n* = 1). The single trovafloxacin DILI case had been reported previously (patient 3 in reference [20]) but was recontacted for the iDILIC study. There were 9 female and 13 male cases. Age range at the time of DILI onset was 20–88 years. All cases were white Europeans resident in the following countries: France (*n* = 2), Italy (*n* = 1), Spain (*n* = 10), Sweden (*n* = 6), United Kingdom (*n* = 3). The most common DILI phenotype was hepatocellular (*n* = 11) but six cases had cholestatic DILI and there were five mixed cases (Table 1). Median latency was 7 days. Most cases (72%) were of at least moderate severity with 27% in the moderate/severe category. There were no severe or fatal cases. Data on the presence of rash, fever, arthralgia and eosinophilia was available for seven cases. Of these, three were positive for rash, five for fever, one for arthralgia and one for eosinophilia suggesting that, apart from fever, hypersensitivity features were not very common (incidence < 50%). When the various clinical characteristics listed in Table 1 were compared between cases due to ciprofloxacin (*n* = 13) and all other FQs (*n* = 9), there were no significant differences between the two groups.

**TABLE 1:** Clinical data summary.

Characteristic	Overall (*n* = 22)	Ciprofloxacin only (*n* = 13)	Other fluoroquinolone^a^ (*n* = 9)
Age (years) (median [range])	66 (20–88)	65 (20–85)	68 (42–88)
Female (%)	9 (41%)	5 (39%)	4 (44%)
Latency (days) (median [range])	7 (1–34)	7 (1–34)	6 (1–14)
Jaundice (%)	15 (68%)	8 (62%)	7 (78%)
Liver biopsy (%)	3 (14%)	1 (8%)	2 (22%)
Death (%)	0	0	0
Hospital admission (%)	17 (77%)	10 (76%)	7 (77%)
ALT (U/L)	358 (63–1626)	331 (117–1559)	384 (63–1626)
ALP (U/L)	208 (75–594)	215 (90–327)	200 (75–594)
Total bilirubin (μmol/L) (median [range])	37 (7–256)	54 (8–240)	20 (7–256)
Pattern of liver injury			
Hepatocellular (%)	11 (50%)	6 (46%)	5 (56%)
Cholestatic (%)	6 (27%)	3 (23%)	3 (33%)
Mixed (%)	5 (23%)	4 (31%)	1 (11%)
Severity			
Mild (%)	6 (27%)	4 (31%)	2 (22%)
Moderate (%)	10 (45%)	5 (39%)	5 (56%)
Moderate/severe (%)	6 (27%)	4 (31%)	2 (22%)
Severe	0	0	0
Fatal	0	0	0
RUCAM score			
Definite or highly probable (score > 8) (%)	3 (14%)	2 (15%)	1 (11%)
Probable (score 6–8) (%)	15 (68%)	10 (77%)	5 (56%)
Possible (score 3–5) (%)	4 (18%)	1 (8%)	3 (33%)

^a^ Other drugs: Levofloxacin 4, moxifloxacin 3, norfloxacin 1, trovafloxacin 1. None of the cases consumed alcohol at above UK safe recommendations. All cases were white Europeans. There were no significant differences in clinical characteristics when cases due to ciprofloxacin were compared with those due to other FQ drugs.

### 3.2 HLA Genotypes

**FIGURE 1:**
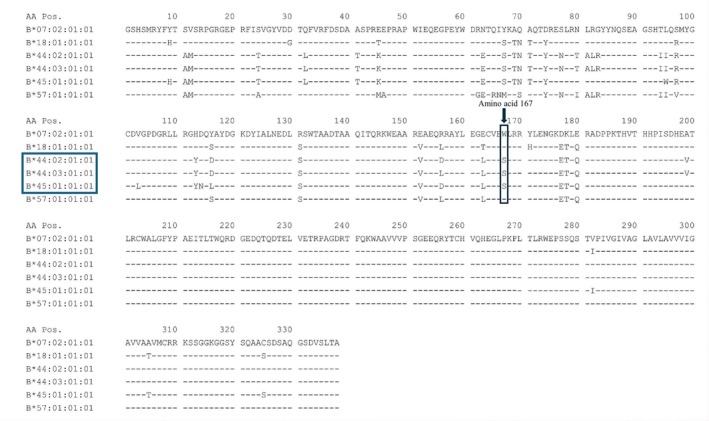
Amino acid sequences of selected HLA‐B alleles. B*07:02 is shown as reference sequence. Sequences of various B*44/*45 alleles together with B*18:01 and B*57:01 are also shown. As indicated, only B*44:02, B*44:03 and B*45:01 have serine (S) at position 167 with the other alleles predicting tryptophan (W) in this position.

**FIGURE 2:**
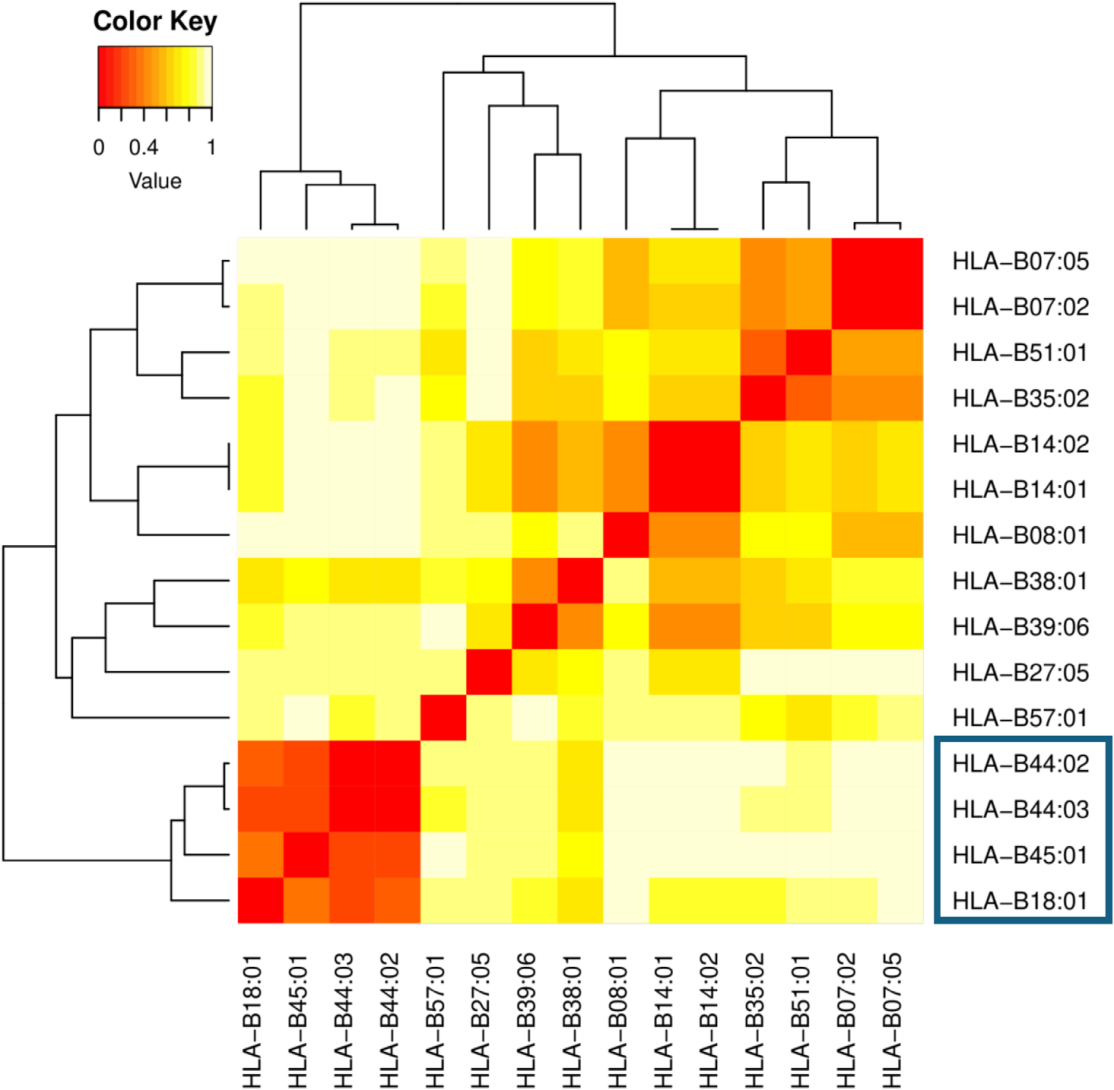
Predicted peptide binding characteristic relationships of selected HLA‐B alleles. Alleles were grouped together based on shared peptide binding profiles, ranging from high (red) to low (yellow). The HLA‐B alleles B*18:01, B*44:02, B*44:03 and B*45:01 (boxed) exhibit similar peptide binding characteristics and form a distinct supertype on this basis.

Table 2 summarises the most significant HLA allele associations comparing cases and controls. These associations were detected for both HLA class I (A, B and C genes) and HLA class II. Complete HLA class I and selected HLA class II genotypes for the cases are shown in Table S2. The most significant findings were associated with the B locus. HLA‐B*44:03 showed a MHC‐wide significantly increased frequency in cases compared to controls (0.20 vs. 0.04), reaching statistical significance after correcting for multiple comparisons (see Section 2.6). The frequency of this allele in DILI controls was comparable to a general European population (0.04 vs. 0.05, Table S3). HLA‐B*44:03 conferred a six‐fold increase in risk of developing FQ‐DILI (OR [95% CI] = 6.01 [2.37–15.28], *p* = 0.0002). Moreover, HLA‐B*44:02, which encodes a protein with strong sequence homology to HLA‐B*44:03 (Figure 1), also appeared more common among cases with an allele frequency of 0.15 compared to 0.08 in controls (OR [95% CI] = 2.86 [1.11–7.34]), with the difference being nominally significant (*p* = 0.03). When any B*44 allele was considered, a strongly significant odds ratio passing Bonferroni correction was seen, showing a five‐fold increase in risk of developing FQ‐DILI (OR [95% CI] = 5.33 [2.45–11.61], *p* = 2.5 × 10^−5^) with cases having double the allele frequency than controls (AF_cases_ = 0.36 and AF_controls_ = 0.12, Table 3). The allele frequency of the two alleles across the European control populations was similar, although less prevalent among Italians (Table S3). Among other alleles with strong peptide binding homology to B*44:03 (Figure 2), HLA‐B*18:01 had a similar frequency in cases and controls (0.04 vs. 0.04) but HLA‐B*45:01, a very rare allele, was enriched in cases (AF_cases_ = 0.05 vs. AF_controls_ = 0.007).

**TABLE 2:** HLA allele associations. Summary statistics of the significant HLA alleles (*p* < 0.05) associated with fluoroquinolone‐induced DILI.

Allele	OR [95% CI]	*p*	AFCA	AFCO
HLA‐B*44:03	6.01 [2.37–15.28]	0.0002	0.20	0.04
HLA‐A*29:02	6.08 [2.12–17.43]	0.0008	0.16	0.03
HLA‐C*16:01	5.21 [1.7–15.97]	0.004	0.14	0.03
HLA‐DQA1*03:01	2.81 [1.36–5.81]	0.005	0.27	0.15
HLA‐DRB1*04:02	6.34 [1.5–26.82]	0.01	0.07	0.01
HLA‐DRB1*04:07	9.97 [1.6–61.99]	0.01	0.05	0.01
HLA‐DRB1*03:02	2.78 [1.22–6.31]	0.01	0.18	0.09
HLA‐B*39:06	7.68 [1.4–42.2]	0.02	0.05	0.01
HLA‐B*44:02	2.86 [1.11–7.34]	0.03	0.16	0.08
HLA‐DRB1*04:01	2.82 [1.07–7.4]	0.03	0.14	0.07

Abbreviations: AFCA, allele frequency in cases; AFCO, allele frequency in controls.

**TABLE 3:** HLA family associations. Summary statistics of the significant HLA family (*p* < 0.05) associated with fluoroquinolone‐induced DILI.

Allele	OR [95% CI]	*p*	AFCA	AFCO
HLA‐B*44	5.33 [2.45–11.61]	2.5 × 10^−5^	0.36	0.12
HLA‐A*29	5.63 [1.98–16.01]	0.001	0.16	0.03
HLA‐DRB1*04	3.22 [1.54–6.73]	0.002	0.27	0.13
HLA‐C*16	4.7 [1.75–12.64]	2.2 × 10^−3^	0.18	0.03
HLA‐DQA1*03	2.81 [1.36–5.81]	0.005	0.27	0.15

Abbreviations: AFCA, allele frequency in cases; AFCO, allele frequency in controls.

The previously reported FQ‐DILI‐associated HLA‐B*57:01 risk allele [15] was only marginally enriched in cases compared to controls (OR [95% CI] = 1.78 [0.46–6.50], AF_cases_ = 0.06 and AF_controls_ = 0.03), not reaching significance (*p* = 0.40).

For HLA‐A, HLA‐A*29:02 showed a significantly increased frequency among the cases (0.16 vs. 0.03, *p* = 0.0008), but no other associations were seen for HLA‐A genes. For HLA‐C, HLA‐C*16:01 was more common among cases (0.14 vs. 0.03).

Among the class II alleles, we found that the cases showed a significantly increased frequency of HLA‐DQA1*03:01 (OR [95% CI] = 2.81 [1.36–5.81], *p* = 0.005, AF_cases_ = 0.27, AF_controls_ = 0.15), confirming its previously reported association with the risk of FQ‐DILI [15]. HLA‐DQA1*03:01 seemed to be more frequent in the Northern European population than in the Southern European population. In our control cohort, the allele frequency was 0.16 among controls from northern Europe, 0.07 from Italy and 0.12 from Spain (Table S3). Additional nominal associations were found with rarer DRB1 alleles such as HLA‐DRB1*04:07, HLA‐DRB1*04:02 and HLA‐DRB1*04:01, which belong to the same allele family. The entire HLA‐DRB1*04 family showed a greater than three times increased risk of FQ‐DILI (OR [95% CI] =3.22 [1.54–6.73]; *p* = 0.002).

We also applied Fisher's Exact Test to have a more conservative analysis due to the limited number of cases, obtaining comparable association results (Table S4).

Conditional analysis on HLA‐B*44 allele family carriage revealed that HLA‐C*16:02 might belong to the same haplotype, but HLA‐B*45:01, HLA‐B*39:06, within the B locus, but also HLA‐A*29:02 and HLA‐DQA1*03:01, were independently associated with the phenotype (Table S5).

The frequency of the most significant HLA alleles in our main analysis was evenly distributed across the causal drugs, confirming that the HLA allele association could be related to the drug class rather than individual drugs (Table S6). Finally, we investigated HLA alleles in the 13 ciprofloxacin only cases, the largest drug‐specific group. This recapitulated the association signals for the B*44 alleles shown in the overall FQ analysis (Table S7).

### 3.3 Amino Acids Associated With HLA Alleles

To further investigate the B*44 association, we analysed polymorphic amino acid residues in the HLA proteins to assess their individual contribution to fluoroquinolone DILI susceptibility. The presence of serine (S) at position 167 in HLA‐B was associated strongly with a significantly increased risk of DILI onset (OR [95% CI] = 6.80 [2.99–15.45], *p* = 4.7 × 10^−6^) but no other strong associations were detected for other amino acids. As summarised in Figure 1, the HLA‐B alleles HLA‐B*44:02, HLA‐B*44:03 and HLA‐B*45:01, but not other alleles including HLA‐B*18:01, HLA‐B*57:01 and HLA‐B*07:02, carry S^167^. In total, 16 of the 22 individuals in the study (73%) are positive for at least one S^167^ residue in their HLA‐B protein based on their genotype (Table S1). The three B alleles positive for S^167^ exhibit a high degree of similarity in peptide binding affinity (Figure 2), along with HLA‐B*18:01, and they are associated with the same supertype B44 [21]. No other B44 supertype alleles were present in the case set. Although classified within the B44 supertype (Figure 2), HLA‐B*18:01 was not associated with FQ‐DILI, having comparable allele frequencies among FQ‐DILI cases, DILI controls, and the broader European population (AF = 0.04). Furthermore, HLA‐B*18:01 did not possess S^167^.

## 4 Discussion

The current study consists of a relatively small cohort of 22 DILI cases but, on the basis of previous reports, this is above the minimum number of 15 cases and 200 controls calculated by others as needed to detect a significant HLA association [22, 23]. A relatively strong HLA association was detected in relation to the B*44 family with 14 of the 22 cases (64%) carrying at least one of these alleles, whereas normally only approximately 12% of Europeans are positive. A large number of drug‐specific HLA associations have now been reported for DILI but this B*44 association is novel. In line with some previous reports and general prescribing data [6, 10, 11, 15], ciprofloxacin is the most common cause of FQ‐DILI in the group but the B*44 association appears to extend to other FQ‐DILI cases, including the now withdrawn trovafloxacin. In addition to the B*44 association, we found a borderline significant risk for B*45:01 as a risk factor in two additional cases. There is strong sequence homology between the B*44 and B*45 alleles, and they are part of the B44 HLA superfamily [21] based on the peptide sequences they recognise. However, importantly, no evidence was found for other HLA alleles such as B*40:01 from the B44 superfamily were risk factors for this type of DILI. This is in line with a recent suggestion that the HLA superfamily concept may not apply well to prediction of drug hypersensitivity reactions [24]. The finding that more than one member of a HLA family contributes to susceptibility to DILI has already been shown for flucloxacillin, where both B*57:01 and B*57:03 are individually overrepresented among DILI cases [25]. This also appears to extend to HLA class II where DRB1*15:02 may be an additional individual risk factor to the well replicated DRB1*15:01 for amoxicillin‐clavulanate DILI [26]. The association seen in the current study with both B*44:02 and B*44:03 is probably detectable because both alleles were observed at comparable frequencies in our study population.

Our findings on HLA genotypes among the cases differ somewhat to those reported recently for a separate FQ‐DILI group from the USA [15]. That study included 44 FQ‐DILI non‐Hispanic white cases which are likely to be comparable genetically to our study population. While our finding of an increased incidence of DQA1*03:01 in the cases (OR 2.81 [1.36–5.81], *p* = 0.005) is very similar to the findings reported for this class II allele in the previous study, we obtained no evidence for a significant alteration in HLA‐B*57:01 frequency among our cases and instead see a highly significant increase in frequency of the HLA‐B*44 family (5.33 [2.45–11.61], 2.5 × 10^−5^), including a strong association with the B*44:03 allele (6.01 [2.37–15.28], *p* = 0.0002). The conditioning analysis indicates that our HLA‐B and HLA‐DQA1 findings are independent so the absence of an association for HLA‐B*44 alleles in the previous study is slightly surprising. One possible explanation could relate to our European DILI population having a different overall allele distribution to the European Americans in the DILIN study. Our finding of a significantly increased FQ‐DILI risk at the amino acid level with S^167^ positivity (odds ratio 6.80 [2.99–15.45], *p* = 4.7 × 10^−6^) is a novel finding. The association of B*44 alleles generally with FQ‐DILI is also novel, though there is a previous report based on HLA serology that B*44 is a risk factor for developing tiopronin‐induced DILI in a Japanese population [27]. That study also reported an association with HLA‐A*33 but this is not the case in the current study and may reflect different HLA haplotypes in the populations being studied.

The clinical features of the current FQ‐DILI cases are in line with previous reports from others with a relatively short time to onset, variable patterns of liver injury and a generally good outcome following drug withdrawal [6, 15]. The limited data available on hypersensitivity features suggests the frequency is slightly higher than that reported for a prospective DILI cohort where it affected 1.5% of patients [28]. The current study and the other recent study [15] both suggest that similar HLA associations with DILI occur across the drug class, which is not necessarily the case for all forms of DILI. The B*44 association we have seen extends to the withdrawn trovafloxacin, with the single case available to us homozygous for B*44:03. Trovafloxacin differs from the other FQs implicated in our DILI cases, as it is more hepatotoxic than other licensed FQs which resulted in market withdrawal in Europe and severe restrictions on use in the USA [29]. While in vitro studies suggest its strong hepatotoxicity may involve a range of effects on gene expression in the liver [29], our data suggests a role also for T cell responses involving HLA. The underlying mechanism for all HLA associations with DILI remains unclear but for a number of causative agents including flucloxacillin, amoxicillin‐clavulanate and green tea extract, in vitro studies suggest inappropriate presentation of chemically modified self peptides to T cells, resulting in local hepatic damage [1, 3]. A similar DILI mechanism seems possible for the various FQs, possibly involving inappropriate recognition of modified self peptides by S^167^ which forms part of pocket A in the HLA protein and recognises the first residue of the peptide being presented [24].

It remains possible that non‐HLA genetic risk factors contribute to the risk of FQ‐DILI. However, SNPs in PTPN22 and ERAP2, which are confirmed risk factors for DILI due to amoxicillin‐clavulanate and likely modulate T cell responses involving HLA, do not similarly increase the risk of FQ‐DILI [14, 30]. In addition, our previous GWAS, which included all the FQ‐DILI cases in the current study, did not detect any FQ‐specific significant signals [13]. Increased numbers of cases would be needed to identify additional risk factors by GWAS or genome sequencing.

Though it was not possible to obtain detailed medical histories from all cases, there was no evidence that any of the patients had suffered other adverse events reported for fluoroquinolones including tendon rupture [31]. Whether there is genetic susceptibility to other adverse events remains unclear.

The study suffers from limitations including the small study group and absence of a replication cohort. Our use of a control group involving DILI cases due to other drugs is another limitation but is a compromise so that appropriate genetic matching of cases and controls can be achieved. We have demonstrated during the study that this matching was successful and confirmed that HLA allele frequencies in our control group are identical to those for European Caucasians reported by others. Our approach of using DILI controls has also been used successfully elsewhere to identify drug‐specific genetic risk factors [32, 33].

Because of concerns about a variety of adverse events linked to this drug class, FQ use in Europe continues to decrease based on regulatory guidelines [34] and makes replication of our current findings more difficult. Nevertheless, the findings are relevant to understanding the underlying mechanisms for DILI and can be used as a basis for further in vitro studies.

## Author Contributions

*Conception and design of the study*: Guruprasad Aithal, Ann Daly, M. Isabel Lucena, Raul Andrade, Mariam Molokhia, Paola Nicoletti. *Collection of clinical data*: Guruprasad Aithal, M. Isabel Lucena, Raul Andrade, Mariam Molokhia, Einar Bjornsson, Dominique Larrey, Mia Wadelius, Par Halberg. *Data analysis*: Paola Nicoletti, Samreen Zafer, Ann Daly. *Drafting of the manuscript*: Ann Daly, Paola Nicoletti. All authors provided comments, revised the draft, and approved the final version of the article.

## Funding

This work was supported by the International Serious Adverse Events Consortium.

## Ethics Statement

The study was conducted in accordance with the principles of the Declaration of Helsinki. The study was approved by the following National and Institutional Review Boards: Ethics Committee at the Virgen de la Victoria University Hospital, Málaga; Regional ethical review board in Uppsala (2008/213 and 2010/231); Ethics Committee of the Medical Faculty of the University of Gothenburg; Leeds East Research Ethics Committee reference 04/Q1206/91; UK REC reference 04/MRE02/70.

## Consent

All participants provided written informed consent.

## Conflicts of Interest

Dr. Nicoletti reports no conflicts of interest for this paper. For full disclosure, she reports consulting agreements with Chiesi Farmaceutici. The other authors declare no conflicts of interest.

## Supporting information


**Figure S1:** Matching genetic ancestry of cases and controls.
**Table S1:** Causative drugs for DILI in the control group.
**Table S2:** HLA genotypes in the cases.
**Table S3:** Allele frequencies of selected HLA alleles across European subpopulations in various control groups.
**Table S4:** Summary statistics of the most significant HLA alleles predisposing to FQ‐DILI risk, using Fisher's exact test.
**Table S5:** Summary statistics of the most significant HLA alleles and family groups from conditional analysis on the presence of HLA‐B*44:03 or HLA‐B*44:02 alleles.
**Table S6:** Allele frequency of the most significant alleles across causal drugs.
**Table S7:** Summary statistics of the nominally significant HLA allele in the ciprofloxacin‐restricted analysis, along with other relevant alleles mentioned in the FQ‐DILI analysis.

## Data Availability

All the data supporting the findings of this study are available within the article and its Supporting Information files or from the corresponding author upon reasonable request.
