# Supplementary material for: HLA‐B*44 Alleles and HLA‐DQA1*03:01 as Genetic Risk Factors for Drug‐Induced Liver Injury due to Fluoroquinolones
Source: Liver Int. 2026 May 15;46:e70699. doi: 10.1111/liv.70699 (PMC13179464; doi:10.1111/liv.70699)
Supplement: Supplementary file 1 — Figure S1: Matching genetic ancestry of cases and controls. Table S1: Causative drugs for DILI in the control group. Table S2: HLA genotypes in the cases. Table S3: Allele frequencies of selected HLA alleles across European subpopulations in various control groups. Table S4: Summary statistics of the most significant HLA alleles predisposing to FQ‐DILI risk, using Fisher's exact test. Table S5: Summary statistics of the most significant HLA alleles and family groups from conditional analysis on the presence of HLA‐B*44:03 or HLA‐B*44:02 alleles. Table S6: Allele frequency of the most significant alleles across causal drugs. Table S7: Summary statistics of the nominally significant HLA allele in the ciprofloxacin‐restricted analysis, along with other relevant alleles mentioned in the FQ‐DILI analysis. [file LIV-46-0-s001.docx]

**HLA-B*44 alleles and *HLA-DQA1*03:01* as genetic risk factors for drug-induced liver injury due to fluoroquinolones**

^1^Paola Nicoletti, ^2,a^M. Isabel Lucena, ^2,a^Raul J. Andrade, ^1^Samreen Zafer, ^3,4^Einar S. Bjornsson, ^5^Pär Hallberg, ^6^Dominique Larrey, ^7^Mariam Molokhia, ^5^Mia Wadelius, ^8^Guruprasad P. Aithal* and ^9^Ann K. Daly*

**Supplementary Figure …………………..……………………………………2**

**Supplementary Tables………............................................................................3**


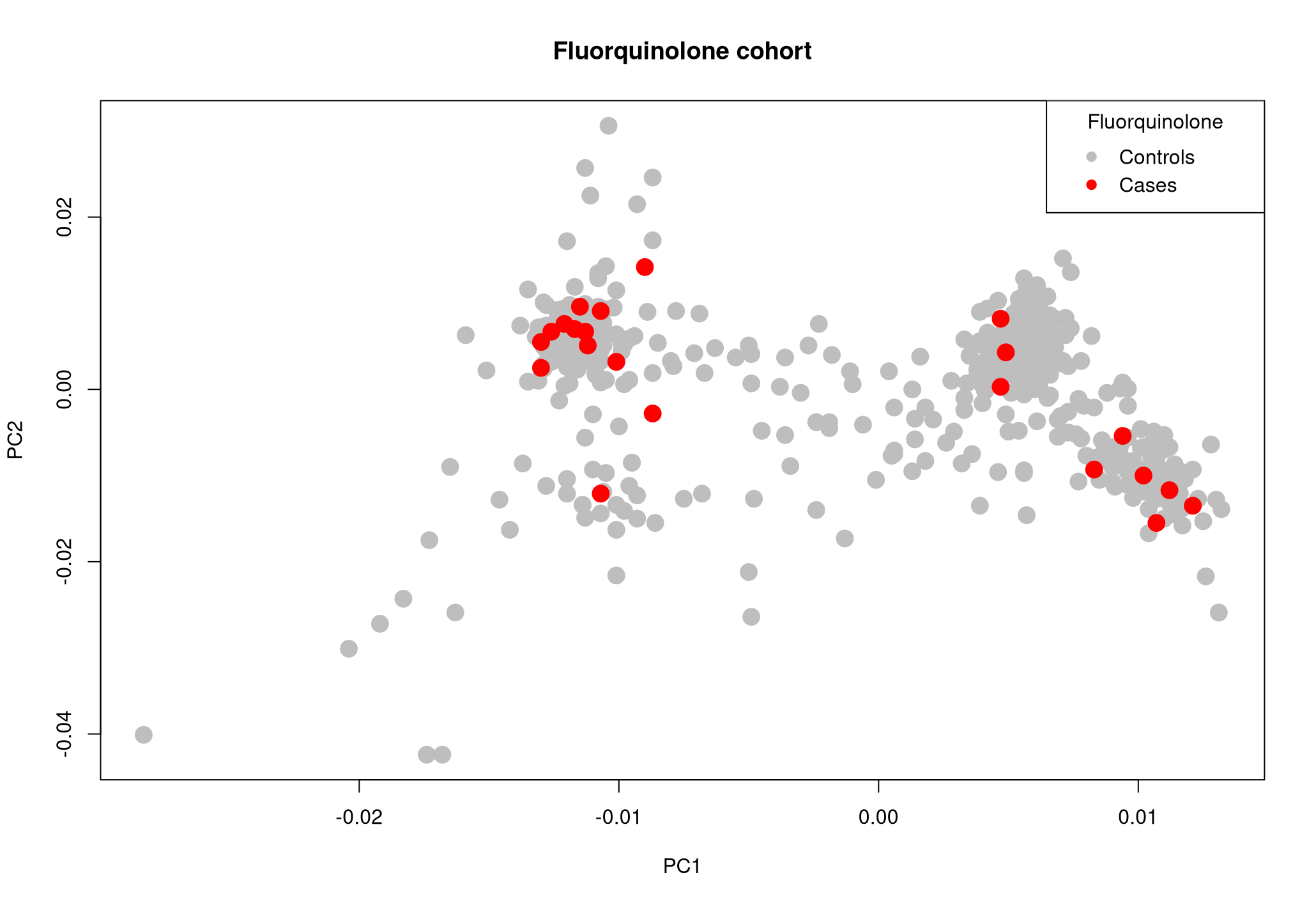


Fluoroquinolone DILI

**Figure S1:** Matching genetic ancestry of cases and controls. Principal components (PCs) were derived for cases and controls. Scatterplots representing the first two principal components of the fluoroquinolone study cohort are shown, confirming the homogenous distribution between cases and controls across major European clusters. Cases are in red, and controls are in grey

**Table S1:** Causative drugs for DILI in the control group

*Aceclofenac, Acetretin, Aciclovir, Adalimumab, Allopurinol, Amoxicillin, Atorvastatin, Azathioprine, Azithromycin, Camellia sinensis, Carbamazepine, Carbimazole, Cefaclor, Cefazolin, Cefotaxim, Cefuroxime, Celecoxib, Cefalexin, Chlorpromazine, Citalopram, Clarithromycin, Cloxacillin, Cyclophosphamide, Cyproterone, Dexibuprofen, Diacerine, Diclofenac, Dicloxacillin, Disulfiram, Doxycycline, Dronedarone, Droxicam, Ebrotidine, Enalapril, Erythromycin, Esomeprazole, Estradiol, Estradiol/levonorgestrel, Etanercept, Ezetimide, Fenofibrate, Flupirtin, Fluvastatin, Gabapentin, Gentamycin, Glucosamine, Ibuprofen, Imatinib, Infliximab, Interferon beta, Isoniazid, Isoniazid/Pyrazinamide, Isoniazid/Pyrazinamide/Rifampicin, Isoniazid/Rifampicin/Pyramizamide/Ethambutol, Isoniazid/Rifampicin, Isotretinoin, Itraconazole, Ketoprofen, Lamotrigine, Lansoprazole, Losartan, Lovastatin, Lymecycline, Medroxyprogesterone, Mercaptopurine, Metamizol, Metandienone, Methimazole, Methotrexate, Milk thistle, Minocycline, Montelukast, Naproxen, Nimesulide, Nitrofurantoin, Olmesartan, Omeprazole, Orlistat, Phenoxymethylpenicillin, Phenprocoumon, Phenytoin, Phyllocontin, Piroxicam, Pravastatin, Propylthiouracil, Ramipril, Rofecoxib, Rosuvastatin, Roxithromycin, Sertraline, Sevoflurane, Simvastatin, Spiramycin/metronidazole, Sulfamethoxazole-trimethoprim, Sulfasalazine, Telmisartan, Terbinafine, Thiamazole, Ticlopidine, Tramadol, Trimethoprim, Valproic acid, Valsartan, Venlafaxine, Warfarin*

The drugs listed above were the likely causes of DILI among the 458 controls. Where drug combinations were used, and the causative drug was unclear, the combination is listed.

**Table S2: HLA genotypes in the cases.**

| **Sample number** | **HLA_A1 (allele 1)** | **HLA_A (allele 2)** | **HLA_B (allele 1)** | **HLA_B (allele 2)** | **HLA_C (allele 1)** | **HLA_C (allele 2)** | **HLA_DRB1 (allele 1)** | **HLA_DRB1 (allele 2)** | **HLA_DQA1 (allele 1)** | **HLA_DQA1 (allele 2)** | **HLA_DQB1 (allele 1)** | **HLA_DQB1 (allele 2)** |
| --- | --- | --- | --- | --- | --- | --- | --- | --- | --- | --- | --- | --- |
| DILI_1 | A*02:01 | A*11:01 | B*35:01 | **B*44:02** | C*04:01 | C*07:04 | DRB1*01:03 | DRB1*04:07 | DQA1*01:01 | DQA1*03:01 | DQB1*03:01 | DQB1*05:01 |
| DILI_2 | A*29:02 | A*29:02 | **B*44:03** | **B*44:03** | C*16:01 | C*16:04 | DRB1*03:01 | DRB1*07:01 | DQA1*02:01 | DQA1*05:01 | DQB1*02:01 | DQB1*02:02 |
| DILI_3 | A*26:01 | A*29:02 | B*27:05 | B*57:01 | C*01:02 | C*06:02 | DRB1*01:03 | DRB1*15:01 | DQA1*01:02 | DQA1*05:01 | DQB1*03:01 | DQB1*06:03 |
| DILI_4 | A*03:01 | A*33:01 | B*14:02 | **B*44:02** | C*05:01 | C*08:02 | DRB1*01:02 | DRB1*04:07 | DQA1*01:01 | DQA1*03:01 | DQB1*03:01 | DQB1*05:01 |
| DILI_5 | A*01:01 | A*03:01 | **B*44:03** | **B*44:03** | C*04:01 | C*06:02 | DRB1*07:01 | DRB1*13:02 | DQA1*01:02 | DQA1*02:01 | DQB1*02:02 | DQB1*06:09 |
| DILI_6 | A*02:01 | A*11:01 | B*45:01 | B*51:01 | C*15:02 | C*16:01 | DRB1*04:02 | DRB1*10:01 | DQA1*01:01 | DQA1*03:01 | DQB1*03:01 | DQB1*05:01 |
| DILI_7 | A*02:01 | A*23:01 | B*35:01 | **B*44:03** | C*04:01 | C*16:01 | DRB1*07:01 | DRB1*14:01 | DQA1*01:01 | DQA1*02:01 | DQB1*02:02 | DQB1*05:03 |
| DILI_8 | A*01:01 | A*32:01 | B*08:01 | B*27:05 | C*02:02 | C*07:01 | DRB1*03:01 | DRB1*04:01 | DQA1*03:01 | DQA1*05:01 | DQB1*02:01 | DQB1*03:02 |
| DILI_9 | A*02:01 | A*02:01 | **B*44:02** | B*51:01 | C*05:01 | C*05:01 | DRB1*04:01 | DRB1*13:01 | DQA1*01:03 | DQA1*03:01 | DQB1*03:02 | DQB1*06:03 |
| DILI_10 | A*02:01 | A*29:02 | B*18:01 | **B*44:03** | C*07:01 | C*16:01 | DRB1*01:03 | DRB1*07:01 | DQA1*01:01 | DQA1*02:01 | DQB1*02:02 | DQB1*05:01 |
| DILI_11 | A*03:01 | A*24:02 | B*39:06 | **B*44:02** | C*05:01 | C*07:02 | DRB1*01:01 | DRB1*13:01 | DQA1*01:01 | DQA1*01:03 | DQB1*05:01 | DQB1*06:03 |
| DILI_12 | A*02:01 | A*68:01 | B*38:01 | **B*44:02** | C*05:01 | C*12:03 | DRB1*04:01 | DRB1*15:01 | DQA1*01:02 | DQA1*03:01 | DQB1*03:02 | DQB1*06:02 |
| DILI_13 | A*01:01 | A*02:01 | B*08:01 | B*57:01 | C*07:01 | C*07:01 | DRB1*03:01 | DRB1*07:01 | DQA1*02:01 | DQA1*05:01 | DQB1*02:01 | DQB1*03:03 |
| DILI_14 | A*01:01 | A*02:01 | **B*44:02** | B*57:01 | C*05:01 | C*06:02 | DRB1*04:02 | DRB1*04:02 | DQA1*03:01 | DQA1*03:01 | DQB1*03:02 | DQB1*03:02 |
| DILI_15 | A*02:01 | A*29:02 | B*14:01 | **B*44:03** | C*08:02 | C*16:01 | DRB1*04:01 | DRB1*07:01 | DQA1*02:01 | DQA1*03:01 | DQB1*02:02 | DQB1*03:02 |
| DILI_16 | A*02:01 | A*02:01 | B*08:01 | B*39:06 | C*07:01 | C*07:02 | DRB1*01:01 | DRB1*03:01 | DQA1*01:01 | DQA1*05:01 | DQB1*02:01 | DQB1*05:01 |
| DILI_17 | A*01:01 | A*02:01 | B*08:01 | B*51:01 | C*07:01 | C*16:02 | DRB1*03:01 | DRB1*16:01 | DQA1*01:02 | DQA1*05:01 | DQB1*02:01 | DQB1*05:02 |
| DILI_18 | A*02:01 | A*23:01 | **B*44:03** | B*51:01 | C*04:01 | C*14:02 | DRB1*07:01 | DRB1*08:01 | DQA1*02:01 | DQA1*04:01 | DQB1*02:02 | DQB1*04:02 |
| DILI_19 | A*02:01 | A*02:01 | B*07:02 | **B*44:02** | C*05:01 | C*07:02 | DRB1*04:04 | DRB1*11:01 | DQA1*03:01 | DQA1*05:01 | DQB1*03:01 | DQB1*03:02 |
| DILI_20 | A*03:01 | A*32:01 | B*07:05 | B*14:02 | C*04:01 | C*08:02 | DRB1*10:01 | DRB1*15:01 | DQA1*01:01 | DQA1*01:02 | DQB1*05:01 | DQB1*06:02 |
| DILI_21 | A*02:01 | A*29:02 | B*18:01 | B*45:01 | C*06:02 | C*07:01 | DRB1*04:01 | DRB1*04:01 | DQA1*03:01 | DQA1*03:01 | DQB1*03:01 | DQB1*03:02 |
| DILI_22 | A*11:01 | A*29:02 | B*38:01 | **B*44:03** | C*07:02 | C*16:01 | DRB1*07:01 | DRB1*13:01 | DQA1*01:03 | DQA1*02:01 | DQB1*02:02 | DQB1*06:03 |

Complete HLA class I genotypes and selected HLA class II genotypes obtained by imputation are shown. B*44 alleles are in bold.

**Table S3:** Allele frequencies of selected HLA alleles across European subpopulations in various control groups.

| **Allele** | **All DILI controls** | **North European DILI controls (n= 308)** | **Spanish DILI controls (n=104)** | **Italian DILI controls (n=30)** | **USA NMDP European cohort**  **(n = 1,242,890)** |
| --- | --- | --- | --- | --- | --- |
| HLA-B*44 | 0.12 | 0.12 | 0.12 | 0.05 | No data available |
| HLA-B*44:02 | 0.07 | 0.08 | 0.06 | 0.03 | 0.09 |
| HLA-B*44:03 | 0.04 | 0.03 | 0.04 | 0.01 | 0.05 |
| HLA-B*44:05 | 0.005 | 0.003 | 0.004 | 0 | 0.003 |
| HLA-B*45:01 | 0.007 | 0.03 | 0.01 | 0 | 0.006 |
| HLA-B*18:01 | 0.05 | 0.04 | 0.07 | 0.06 | 0.04 |
| HLA-DQA1*03:01 | 0.15 | 0.17 | 0.12 | 0.05 | No data available |

Abbreviations: DILI, Drug-Induced Liver Injury; USA NMDP, USA National Marrow Donor Program

**Table S4:** Summary statistics of the most significant HLA alleles predisposing to FQ-DILI risk, using Fisher's exact test.

| **Allele** | **OR [95%CI]** | **P** |
| --- | --- | --- |
| HLA-B*44 | 4.28 [2.24-8.16] | 4.15E-05 |
| HLA-B*44:03 | 6.88 [3.06-15.48] | 5.60E-05 |
| HLA-C*16 | 6.14 [2.64-14.27] | 0.0003 |
| HLA-A*29:02 | 6.23 [2.55-15.23] | 0.0006 |
| HLA-A*29 | 5.79 [2.38-14.07] | 0.0008 |
| HLA-C*16:01 | 5.2 [2.03-13.34] | 0.003 |
| HLA-DRB1*04:02 | 9.5 [2.37-38.08] | 0.009 |
| HLA-DRB1*04 | 2.49 [1.25-4.96] | 0.01 |
| HLA-DQA1*03:01 | 2.15 [1.08-4.28] | 0.03 |
| HLA-DQA1*03 | 2.15 [1.08-4.28] | 0.03 |
| HLA-DRB1*04:07 | 7.22 [1.42-36.86] | 0.05 |
| HLA-DRB1*03:02 | 2.29 [1.03-5.1] | 0.06 |
| HLA-B*39:06 | 6.18 [1.25-30.68] | 0.06 |
| HLA-B*44:02 | 2.29 [0.98-5.32] | 0.08 |
| HLA-DRB1*04:01 | 1.97 [0.8-4.82] | 0.14 |

**Table S5:** Summary statistics of the most significant HLA alleles and family groups from conditional analysis on the presence of HLA-B*44:03 or HLA-B*44:02 alleles

| **allele** | **OR [95%CI]** | **P** |
| --- | --- | --- |
| HLA-B*45 | 13.95 [2.19-89.01] | 0.005 |
| HLA-B*45:01 | 13.95 [2.19-89.01] | 0.005 |
| HLA-DRB1*04 | 3.01 [1.38-6.53] | 0.005 |
| HLA-DRB1*04:07 | 11.6 [1.68-80.15] | 0.01 |
| HLA-DQA1*03 | 2.59 [1.2-5.57] | 0.01 |
| HLA-DQA1*03:01 | 2.59 [1.2-5.57] | 0.01 |
| HLA-DRB1*03:02 | 2.92 [1.23-6.94] | 0.01 |
| HLA-A*29:02 | 4.15 [1.28-13.41] | 0.02 |
| HLA-A*29 | 3.86 [1.21-12.34] | 0.02 |
| HLA-B*39:06 | 9.14 [1.3-64.24] | 0.03 |
| HLA-DRB1*04:02 | 5.82 [1.2-28.08] | 0.03 |

**Table S6:** Allele frequency of the most significant alleles across causal drugs

| **HLA allele** | **Drug** | **AF** | **N** |
| --- | --- | --- | --- |
| HLA-B*44:02 | Ciprofloxacin | 0.19 | 13 |
|  | Levofloxacin | 0.13 | 4 |
|  | Moxifloxacin | 0.17 | 3 |
|  | Norfloxacin | 0 | 1 |
|  | Trovafloxacin | 0 | 1 |
| HLA-B*44:03 | Ciprofloxacin | 0.19 | 13 |
|  | Levofloxacin | 0.13 | 4 |
|  | Moxifloxacin | 0.17 | 3 |
|  | Norfloxacin | 0 | 1 |
|  | Trovafloxacin | 1 | 1 |
| HLA-B*45:01 | Ciprofloxacin | 0.04 | 13 |
|  | Levofloxacin | 0.1 | 4 |
|  | Moxifloxacin | 0 | 3 |
|  | Norfloxacin | 0 | 1 |
|  | Trovafloxacin | 0 | 1 |
| HLA-DQA1*03:01 | Ciprofloxacin | 0.23 | 13 |
|  | Levofloxacin | 0.5 | 4 |
|  | Moxifloxacin | 0.17 | 3 |
|  | Norfloxacin | 0.5 | 1 |
|  | Trovafloxacin | 0 | 1 |

AF = allele frequency

**Table S7:** Summary statistics of the nominally significant HLA allele in the ciprofloxacin-restricted analysis, along with other relevant alleles mentioned in the FQ-DILI analysis.

| **allele** | **OR[95%CI]** | **P** | **AFCA** | **AFCO** |
| --- | --- | --- | --- | --- |
| HLA-B*44 | 6.08 [1.73-21.4] | 0.0003 | 0.385 | 0.363 |
| HLA-B*44:03 | 6.53 [1.85-23.04] | 0.001 | 0.192 | 0.204 |
| HLA-B*39:06 | 4.52 [1.36-15.09] | 0.003 | 0.077 | 0.045 |
| HLA-A*29:02 | 5.37 [1.25-23.06] | 0.004 | 0.154 | 0.159 |
| HLA-A*29 | 46.19 [2.25-947.7] | 0.005 | 0.154 | 0.159 |
| HLA-C*16:04 | 14.97 [2.51-89.13] | 0.01 | 0.038 | 0.001 |
| HLA-C*16 | 6.42 [2.35-17.49] | 0.01 | 0.154 | 0.182 |
| HLA-C*16:01 | 3.43 [1.1-10.69] | 0.02 | 0.115 | 0.136 |
| HLA-B*44:02 | 6.93 [2.14-22.49] | 0.03 | 0.190 | 0.159 |
| HLA-DQA1*03:01 | 2 [0.76-5.27] | 0.1599 | 0.230 | 0.272 |
| HLA-B*18:01 | 0.79 [0.1-6.32] | 0.8246 | 0.040 | 0.047 |
| HLA-B*45:01 | 6.32 [0.66-61.06] | 0.1109 | 0.040 | 0.008 |

AFCA = allele frequency in cases; AFCO = allele frequency in controls
